# The Essay on Medical Pneumatology, a Physiological, Clinical and Therapeutic Investigation of the Gases

**Published:** 1890-02

**Authors:** 


The Essay on Medical Pneumatology, a Physiological, Clinical and Therapeutic Investigation of the Gases, by J. N. Demarquay, translated, with Notes, Additions and Omissions by Samuel S. Wallian, is before me.

A book comprising about 300 pages, and replete with a most thorough scientific investigation of the therapeutic value of pure oxygen gas. Since the publication of Demarquay's work in 1866, the medical world have steadily increased in the use of oxygen as a therapeutic agent; and now that the translation, with his notes, comments and additions, so beautifully and with such perfect expression, and with the very essence of experience founded upon careful manipulation and experiment, comes to the front with this proof from his more recent investigation, it is only easy and natural to be persuaded of a day in the near future when every truly scientific practitioner of medicine and surgery will be equipped with apparatus for the production and administration of this valuable agent. His experiments clearly define the condition of oxygen diagnosis, and sums up such physiological conditions as are more susceptible to permanent relief; also gives the most perfect chemical formula for the production as well as the latest and best apparatus for administration. He not only produces cuts of a most perfect and easy apparatus for office use, but those of such convenient construction as to be carried in the foot of the physician's buggy that the patient in bed can as readily have the benefit of pure oxygen as those able to visit the office. In short, it is a work covering the ground up to the present day. S. G. H.

